# The causal effect of childhood measles vaccination on educational attainment: A mother fixed-effects study in rural South Africa

**DOI:** 10.1016/j.vaccine.2015.04.072

**Published:** 2015-09-11

**Authors:** Tobenna D. Anekwe, Marie-Louise Newell, Frank Tanser, Deenan Pillay, Till Bärnighausen

**Affiliations:** a USDA Economic Research Service, Washington, DC 20224, USA; b Wellcome Trust Africa Centre for Health and Population Studies, Mtubatuba 3935, South Africa; c University of Southampton, Southampton SO17 1BJ, UK; d University College London, London WC1E 6BT, UK; e Harvard T.H. Chan School of Public Health, Boston, MA 02115, USA

**Keywords:** CI, confidence interval, DSA, demographic surveillance area, DTP, diphtheria–tetanus–pertussis vaccine, DTP3, complete series of three DTP doses, FE, fixed effects, HDSS, health and demographic surveillance system, MDG, Millennium Development Goal, SD, standard deviation, Childhood measles vaccination, Educational attainment, Mother fixed-effects study

## Abstract

**Background:**

Because measles vaccination prevents acute measles disease and morbidities secondary to measles, such as undernutrition, blindness, and brain damage, the vaccination may also lead to higher educational attainment. However, there has been little evidence to support this hypothesis at the population level. In this study, we estimate the causal effect of childhood measles vaccination on educational attainment among children born between 1995 and 2000 in South Africa.

**Methods and findings:**

We use longitudinal data on measles vaccination status and school grade attainment among 4783 children. The data were collected by the Wellcome Trust Africa Centre Demographic Information System (ACDIS), which is one of Africa's largest health and demographic surveillance systems. ACDIS is located in a poor, predominantly rural, Zulu-speaking community in KwaZulu-Natal, South Africa. Using mother fixed-effects regression, we compare the school grade attainment of siblings who are discordant in their measles vaccination status but share the same mother and household. This fixed-effects approach controls for confounding due to both observed and unobserved factors that do not vary between siblings, including sibling-invariant mother and household characteristics such as attitudes toward risk, conscientiousness, and aspirations for children. We further control for a range of potential confounders that vary between siblings, such as sex of the child, year of birth, mother's age at child's birth, and birth order. We find that measles vaccination on average increases school grade attainment by 0.188 grades (95% confidence interval, 0.0424–0.334; *p* = 0.011).

**Conclusions:**

Measles vaccination increased educational attainment in this poor, largely rural community in South Africa. For every five to seven children vaccinated against measles, one additional school grade was gained. The presence of a measles vaccination effect in this community is plausible because (i) measles vaccination prevents measles complications including blindness, brain damage, and undernutrition; (ii) a large number of number of children were at risk of contracting measles because of the comparatively low measles vaccination coverage; and (iii) significant measles transmission occurred in the community where this study took place during the study observation period. Our results demonstrate for the first time that measles vaccination affects human development not only through its health effects but also through its effects on education.

## 1 Introduction

Childhood vaccinations are among the most cost-effective public health interventions [1], [2], yet coverage for several vaccinations is far from universal in many countries [3]. In this paper, we focus on measles vaccination for several reasons. Measles vaccination coverage remains low in sub-Saharan Africa; in many countries in the region [4], coverage is far below the 93–95% required for herd immunity [5]. For instance, measles vaccination coverage (among one-year-olds) in South Africa is only 66% [6], in the WHO African Region only 73% [7], and in KwaZulu-Natal, South Africa (where this study took place), overall 77% but with significant variation in coverage across communities [8]. As a result, populations in South Africa are vulnerable to measles, and endemic measles transmission and measles outbreaks have been common [9], [10], [11]. Measles kills more people in the WHO African Region than any other vaccine-preventable disease such as pertussis, diphtheria, or tetanus (36,900 children under five each year) [12].

Vaccinations improve health outcomes, but it is also plausible that they improve *non-health* outcomes via those health improvements [13], [14]. By warding off diseases and subsequent complications (such as undernutrition, blindness, and encephalitis), vaccinations may protect children's cognitive and physical development, which in turn enhances their capacity for educational attainment, labor market productivity, harnessing life chances, and fulfilling social roles [15], [16], [17], [18], [19].

Educational attainment is a particularly important potential outcome of measles vaccination. Education is a human right [20], [21]; it is also essential for human development, as it expands people's life chances and capabilities [22]. Furthermore, education is instrumentally important because it can improve labor productivity, which fuels economic development [23], [24], [25]. The importance of education is underscored by the fact that the United Nations Development Programme has for decades been using educational attainment as one of the three components of its Human Development Index [26], [27]. Despite the fundamental importance of education for people and societies, average educational attainment in many places remains low. For instance, educational attainment in South Asia and sub-Saharan Africa is less than five years [26]—well below the level of secondary school, which begins at grade seven in many countries [28].

Despite the fact that measles vaccination prevents a variety of disabling health conditions that are likely to affect educational attainment (e.g., blindness [29], measles encephalitis and subsequent neurological damage [30], [31], and child undernutrition [30], [32], [33]) and the general interest in vaccination effects on education [15], no study has to date tried to establish the effect of measles vaccination on educational attainment. With this study, we aim to close this evidence gap. We estimate the causal effect of childhood measles vaccination on educational attainment among children born between 1995 and 2000 in a poor, predominantly rural community in KwaZulu-Natal, South Africa.

## 2 Methods

### 2.1 Data sources and samples

We used longitudinal data from a health and demographic surveillance system (HDSS) in rural KwaZulu-Natal, South Africa, that was established and is maintained by the Wellcome Trust-funded Africa Centre for Health and Population Studies. The HDSS started in 2000 and covers a demographic surveillance area (DSA) of 438 square kilometers near the market town of Mtubatuba in the predominantly rural Umkhanyakude district of KwaZulu-Natal. The surveillance system covers the entire population of about 85,000 Zulu-speaking people who are members of the 11,000 households in the DSA. Most households are multi-generational, and average household size is 7.9 (SD = 4.7) members. Although this is a predominantly rural area, the principal source of income for most households is waged employment and state pensions rather than agriculture. In 2006, approximately 77% of households in the surveillance area had access to piped water and toilet facilities [34]. Due to the availability of antiretroviral therapy in South Africa's public-sector health system starting in 2004, adult life expectancy in this community increased from about 49 years in 2003 to 61 years in 2011 [35].

Data on all births in the year of a household's first interview in the surveillance as well as the previous five years were elicited from all women residing in the DSA. For each child, childhood vaccination data were elicited. We measured the outcome, school grade attainment, up to the year 2007. Our sample for this study consists of all children who were born between 1995 and 2000 and were members of households residing in the DSA in 2007. 1995 was the first year that childhood vaccination data became available in the HDSS; the year 2000 cutoff ensures that every child had the chance to complete at least one year of school by 2007 in longitudinal follow-up.

In the sample for complete-case analysis, the total number of children was 4783 and the total number of mothers was 4080. In the sample for multiple-imputation analysis, the total number of children was 7509 and the total number of mothers was 6148. Even though the main effect estimate in our fixed-effects models is based only on the comparison of children who share the same mother but differ in their measles vaccination status (607 in the complete-case analysis and 1031 in the multiple-imputation analysis), we kept all other children in the sample for analysis, because these observations contribute to the estimation of the regression constant and the *R*^2^ statistic without affecting the size or significance of the measles effect estimate.

The surveillance questionnaires and descriptions of the data sets are available on the website of the Africa Centre for Health and Population Studies (http://www.africacentre.ac.za).

### 2.2 Exposure and outcome variables

#### 2.2.1 Vaccination status

Our exposure variable is measles vaccination status at 12 months of age. A child was coded as either vaccinated or unvaccinated for measles by 12 months of age. We coded a child as having received his or her measles dose by 12 months of age if at least one of the following two conditions was met: first, the national vaccination card (the so-called Road-to-Health card) was the data source and the date of vaccination dose was within one year of birth or, second, mother's report was the data source and indicated that the child had received the vaccination within one year of birth. Mother's report of her children's vaccination status has been validated in this community by Ndirangu et al. [36]. If vaccination card information and mother's report were both available, we used the card information. This approach to coding vaccination data is the same that is used in many other population-based surveys, such as the Demographic and Health Surveys (DHS) [37]. Children with missing card information and missing mother's report and children with missing covariate information were excluded in the complete-case analyses. To test the robustness of our findings to missing observations, we multiply imputed vaccination status and other missing data and repeated the analyses with the imputed datasets [38]. The sample size for the main, complete-case analysis was 4783; the sample size for the analysis of the multiply imputed data was 7509.

#### 2.2.2 Educational attainment

To capture educational attainment, we used the highest school grade that a child had attained at the last HDSS household interview up to the year 2007. Not all children were eligible for outcome measurement in 2007 (e.g., because their families had out-migrated). To ensure the comparability of school grade outcomes between children who were born in the same year but had their school grade measured in different years, we controlled for a child's age at start of the school year in which the household interview was conducted. Also, to ensure the comparability of schooling outcomes between children who were born in different calendar years (and who would therefore be expected to have different levels of school grade attainment in later calendar years), we controlled for year of birth. We excluded a small number of children (49, or 1.0% of the complete-case analysis sample) who had implausible reported grade levels. We defined “implausible reported grade level” as three or more grades ahead of the grade that a child would have attained had she started school (grade 1) at age seven and advanced by one grade per year. (According to the South African Schools Act of 1996 [39], [40], children must start school no later than the calendar year in which they turn seven years old.) At the time of the last measurement of the outcome in this study (school grade attainment), the children were aged six to eleven years.

### 2.3 Statistical analysis

We estimate the effect of childhood measles vaccination on educational attainment using mother fixed-effects analysis. The mother fixed effects control for all observed and unobserved factors that are shared by siblings, including mother and household characteristics that do not vary between siblings, such as risk attitudes, conscientiousness, and aspirations for children's futures. We also control for a number of factors that can vary between siblings: sex, age at start of the school year in which the household interview was conducted, calendar year of birth, mother's age at child's birth, and birth order. Finally, in separate analyses, we additionally control for the number of doses of diphtheria–tetanus–pertussis vaccine (DTP) received. DTP is often used as a proxy for immunization system performance [41]. Here, DTP coverage serves as a powerful control variable to account for any potential sibling-varying confounding that is related to differences between siblings in access to vaccinations in general that are not captured by the other sibling-varying control variables. These sibling-varying confounding factors include differential availability of vaccination between siblings that is not already captured by the calendar-year control variables (e.g., when a family moves their home closer to a vaccination clinic and at the same time moves closer to the nearest school). They also include changes in maternal and paternal knowledge, attitudes, and behaviors that can affect vaccination and school grade attainment.

The mother fixed-effects regression has the form:

(1) $$Y_{im}=\alpha +\beta V_{im}+\gamma X_{im}+\lambda X_{m}+\mu_{m}+\varepsilon_{im}$$

where *Y*_*im*_ is the school grade of child *i* with mother *m*. *V*_*im*_ is child *i*'s measles vaccination status at 12 months of age. *β* is the main parameter of interest in this study: the conditional association between childhood measles vaccination and school grade attainment. *X*_*im*_ is a vector of child *i*'s characteristics, and *X*_*m*_ is mother *m*'s age at the time of child *i*'s birth. *μ*_*m*_ is the mother fixed effect and ɛ_*im*_ is the error term.

We performed four regression analyses: complete-case analyses with and without the DTP covariates and analyses using multiple imputation of missing data, again both with and without DTP covariates. Some data were missing for four of the variables we use in the regression analyses: school grade attainment (missing for 69 of 7509 observations), measles vaccination (missing for 100 observations), DTP vaccination (missing for 2600 observations), and birth order (missing for 117 observations). We carried out 40 imputations in the multiple imputation, which exceeds the commonly recommended minimum numbers of imputations [42] but is unproblematic given today's computing power.

In all models, we clustered heteroskedasticity-robust standard errors at the level of the mother to account for correlation in outcomes among children who share the same mother. Analyses were conducted using Stata version 11 (StataCorp LP, College Station, TX).

## 3 Results

Table 1 presents descriptive statistics for both the analyses using the complete-case sample and the analyses after multiple imputation. Measles vaccination coverage at 12 months of age was 66% in the complete-case analysis and average school grade attainment was 2nd grade (Table 1). On average, children were about one year behind the school grade attainment they would be expected to have reached by the time of the last school grade assessment in this study, had they progressed through the grades without delay since entering school. Table 2 presents the estimates of the causal effect of measles vaccination on school grade attainment, controlling for mother fixed effects (FE). Our results show that childhood measles vaccination significantly increases school grade attainment: children vaccinated against measles by 12 months of age benefit on average by an additional 0.188 years of school grade attainment (in the complete-case analyses) and by an additional 0.143–0.149 years of school grade attainment (in the analyses after multiple imputation), compared to siblings who were not measles-vaccinated by 12 months of age. In other words, for every five to seven children receiving the measles vaccination, we would expect to gain one full year of school grade attainment among children aged between six and eleven years. When we add DTP vaccination status as an additional control variable, the results remain essentially unchanged.

We also performed a sensitivity analysis to test whether our findings were robust when we included in the treatment group those children who received measles with a one-month delay (i.e., between 12 and 13 months). The results of this analysis are essentially the same as those of the main analysis: The measles effect size estimate was 0.176 [*p*-value 0.020; 95% CI = (0.028, 0.324)] in the complete-case analysis without DTP3 covariate; 0.171 [*p*-value: 0.051; 95% CI = (−0.0006, 0.342)] in the complete-case analysis with DTP3 covariate; 0.130 [*p*-value: 0.019; 95% CI = (0.021, 0.239)] in the multiple-imputation analysis without DTP3 covariate; and 0.114 [*p*-value: 0.102; 95% CI = (−0.023, 0.251)] in the multiple-imputation analysis with DTP3 covariate.

Taking an estimate of an 11.7% gain in wages per year of schooling in sub-Saharan Africa [43] and the measles vaccination effect on years of schooling established in this study (0.188), we estimate that a child who is currently not vaccinated against measles would be expected to gain a more than 2% wage increase per year from measles vaccination.

## 4 Discussion

We find that measles vaccination significantly increases educational attainment in a predominantly rural community in South Africa with comparatively low measles vaccination coverage. Our results indicate that for every five to seven children vaccinated against measles by 12 months of age, one additional year of schooling is gained. Based on estimated wage returns to education [43], this effect translates into an annual wage gain of more than 2% due to measles vaccination. Because the children in this sample are quite young (six to eleven years of age), the final schooling deficit among the unvaccinated may be even larger than that found in this study.

Our finding that measles vaccination substantially affects children's school grade attainment is plausible. Measles vaccination prevents blindness, which can severely impede educational attainment, particularly in settings where schools for the blind are not available [29]. Measles vaccination can also prevent measles encephalitis and its sequelae, which include neurological damage [30], [31]; middle ear infections, which can lead to hearing impairment and scholastic underperformance [44]; and child undernutrition [30], [32], [33], which predicts lower educational attainment and worse academic performance in developing countries [45], [46]. Furthermore, infections and undernutrition in children under five can cause general malaise, apathy, and decreased physical activity and play [47], [48]; young children with these symptoms generally receive less stimulation from adults and fewer learning opportunities, which can negatively impact their cognitive and physical development [48].

The educational effect of measles vaccination is likely to be relatively large in poor communities, such as the one where this study took place, because high underlying levels of undernutrition weaken the immune system and exacerbate the severity of measles disease and measles complications [49], [50]. The educational effect of measles vaccination may also be more pronounced in communities where assistive technology and special education for the disabled [51] are largely lacking, as is the case in the study community. In South Africa in general, students with learning disabilities and other special needs face major barriers to educational access and performance: many schools lack the infrastructure and resources to accommodate students with disabilities, and educators are not trained or equipped to work with students with disabilities in the classroom. A student's failure to master the curriculum can result in grade repetition, which is a major cause of over-age enrollment in South Africa's schools. Over-age enrollment and special educational needs have been identified as risk factors for dropping out of school in South Africa [52].

A recent study [8] found children aged 12–23 months in this community had 77% measles vaccination coverage in 2006—far below the herd immunity threshold (93–95%), thus leaving the community vulnerable to significant measles incidence and outbreaks. Indeed, data from the South African national epidemiological reporting system show significant measles transmission during the period of the present study [10], [11]. Although we do not have information on the incidence of measles infection and complications for children in our particular sample, it seems likely that a number of children contracted measles throughout the period 1995 through 2007 due to the low measles vaccination coverage rates and the epidemiological evidence of continued measles transmission [53].

The causal effect of measles vaccination on educational attainment can never be estimated in a randomized experiment, because it is obviously unethical to withhold a vaccination of proven effectiveness and safety. When randomized experiments are not possible, quasi-experimental studies are a powerful alternative for strong causal evaluation. We thus use a quasi-experimental approach (mother fixed-effects analysis) to estimate the effect of childhood measles vaccination on educational attainment. The identifying assumption of this analysis is that vaccination status among siblings is as good as randomly assigned after controlling for all sibling-invariant mother and household characteristics and several sibling-varying factors that might confound the relationship between sibling vaccination status and educational attainment (e.g., birth cohort, mother's age at birth, and birth order). We also control for the sibling-varying factor DTP vaccination, a proxy for DTP coverage that powerfully controls for potential sibling-varying confounding factors that determine vaccination status in general rather than specifically for measles.

Outside of a randomized controlled trial, a mother fixed-effects study that controls for a range of potentially important sibling-varying confounders is among the strongest types of causal inference strategies available to answer the question of whether a childhood vaccination has an effect on educational attainment [54]. One likely reason why such a quasi-experimental study has not been previously carried out is that such studies need very large sample sizes in order to have sufficient power to detect significant effects. Our sample size here is large and proved large enough to detect a highly significant measles vaccination effect on educational attainment in a rural community in South Africa where measles vaccination coverage is overall low. This data analytical opportunity arose because we had access to a large longitudinal, population-based dataset, which includes all children born from 1995 through 2000 to mothers who lived in a community of about 85,000 people in 2000. Measles vaccination coverage in the community is comparatively low, and our dataset included an overall large number of those children who contribute to the effect size estimation in mother fixed-effects analysis: children who have at least one measles vaccination-discordant sibling.

Our study has several limitations. A first limitation is that, given our identification strategy, we do not capture the herd effects of measles vaccination on educational attainment (i.e., that some unvaccinated children may benefit in their educational attainment because of the vaccination of others). The full causal impact of measles vaccination on educational attainment in the community is thus likely larger than the effect size we have estimated in this study, and future studies estimating the social value of measles vaccination should treat this effect size as a lower bound of the true educational benefit of measles vaccination [55]. A second limitation is that we do not have information on children's HIV status. HIV-positive children may be less likely to receive vaccination (e.g., because of illness or stigma) [8] and less likely to perform well in school (e.g., because of HIV-related diseases). Similarly, because of HIV stigma, an HIV-infected child within the family may have adverse consequences on siblings, including access to immunization. However, antiretroviral treatment did not become available in this community until the end of 2004 and did not reach significant population coverage until the end of 2006 [56]. It is thus unlikely that many children in our sample (born 1995–2000) who were infected in the perinatal period survived to school age [57]. In addition, as sexual debut is relatively late in South Africa, it is further unlikely that a significant number of children in the sample were infected through sexual transmission. According to a nationally representative study in 2002, in the age group 12–14 years, only 1.9% of males and 1.5% of females reported having had sex [58]. Finally, only a very small proportion of children who were infected with HIV through sexual transmission would have developed HIV-related symptoms by the time their school grade attainment was observed in this study, due to the long latency of HIV [59].

A third limitation is the possibility that disease may have caused children to both miss vaccinations at the clinic and do poorly in school. However, since in this study we observe measles vaccination exposures and school grade outcomes six to ten years apart, only childhood diseases (or disease symptoms) that last many years without causing death could have had such a confounding effect, and such diseases are overall rare. A fourth limitation is that our fixed-effects model relies on vaccination status-discordant siblings (i.e., at least one sibling is vaccinated and at least one other is unvaccinated) and is therefore unable to examine the causal effect of measles vaccination on educational attainment among families with only one child. Consequently, our estimates may be generalizable only to families with two or more children. Furthermore, because our identification strategy relies on households where at least one sibling is vaccinated and at least one sibling is unvaccinated, and because of cross-sibling herd effects [60], an unvaccinated sibling in our study is partially protected from measles infection by his or her sibling's measles vaccination. In other words, our control group is partially protected from measles. This means that our estimated effect size is a lower bound on the true effect size for children in multi-child families where none of the children are vaccinated against measles.

Results from a sensitivity analysis of delayed measles vaccination (by 1 month) support our main findings. It is important to note that as measles vaccination is increasingly delayed the risk of acquiring measles increases. In contexts where measles vaccination coverage remains relatively low, such as in rural KwaZulu-Natal, the impact of “catch-up” and “mop-up” [61] campaigns is an important policy question. Future research in areas where there has been substantial variation in such strategies could follow our basic analytical approach here to establish campaign impact on educational attainment [61], [62], [63].

In order to better understand how vaccine-preventable disease is linked to educational attainment, we also suggest that future research use child-level data to examine the relationships between childhood infectious disease and subsequent complications (occurrence, timing, type, and severity), entry into school (late, on-time, and early), and grade progression (slow, normal, and fast). Such detailed analyses could establish the causal pathways from measles vaccination to educational attainment. Although we argue in this paper that measles and its sequelae mediate the relationship between measles vaccination status and educational attainment, our data do not permit us to investigate this question directly.

The returns to primary education in developing countries are very high. In fact, both the social and private returns to primary education are substantially higher than those to secondary or tertiary education, and they are higher in developing countries than in developed countries [43]. In this study, we have therefore focused on the causal effect of measles vaccination on children's educational attainment in primary school. In future work, we plan to extend this analysis to also study the effect of childhood measles vaccination on education and labor market outcomes in older ages.

Achieving universal coverage with vaccinations of proven effectiveness is desirable for many reasons, foremost to save lives and prevent disease. Because global child mortality could be substantially reduced if all children received the measles vaccine, the fourth Millennium Development Goal (MDG) calls for reduction of child mortality to be achieved in part by increasing the proportion of one-year-old children immunized against measles. But our findings suggest that measles vaccination would also boost progress toward the second MDG, which calls for universal primary education. Finally, because education can promote the development of productive and innovative adults, higher measles vaccination coverage rates could accelerate nations’ human and economic development.

## Financial disclosure

We are grateful for funding from GAVI's PneumoADIP (Pneumococcal Vaccines Accelerated Development and Introduction Plan) at Johns Hopkins Bloomberg School of Public Health through the grant “Benefit-cost analyses for vaccination against pneumococcus, rotavirus, *Haemophilus influenzae* type B, and other vaccine-preventable diseases.” The Africa Centre Demographic Information System was supported by Wellcome Trust grant no. 65377 to the Africa Centre for Health and Population Studies. The funders had no role in study design, data collection and analysis, decision to publish, or preparation of the manuscript.

## Figures and Tables

**Table 1:** Descriptive statistics.

	Complete-case analysis	Multiple-imputation analysis
Sample size	4783	7509
School grade, mean (SD)		
Grade	2.3 (1.6)	2.4 (1.7)
Grade-for-age	−0.98 (1.1)	−1.00 (1.1)
Measles vaccination at 12 months of age, % (SD)		
No	34.4 (47.5)	25.4 (43.5)
Yes	65.6 (47.5)	74.6 (43.5)
Sex, % (SD)		
Female	49.7 (50.0)	50.2 (50.0)
Male	50.3 (50.0)	49.8 (50.0)
Age,^a^ % (SD)		
6	17.8 (38.3)	17.5 (38.0)
7	18.0 (38.4)	16.8 (37.4)
8	18.9 (39.2)	17.6 (38.1)
9	18.5 (38.9)	18.0 (38.4)
10	15.0 (35.7)	16.0 (36.6)
11	11.7 (32.2)	14.1 (34.8)
Birth cohort, % (SD)		
1995	13.7 (34.3)	16.6 (37.2)
1996	16.8 (37.4)	17.6 (38.1)
1997	19.5 (39.7)	19.1 (39.3)
1998	19.4 (39.5)	17.6 (38.0)
1999	16.4 (37.0)	15.0 (35.8)
2000	14.2 (34.9)	14.1 (34.8)
Mother's age at child's birth (years), % (SD)		
<18	7.8 (26.9)	8.7 (28.2)
18–19	9.8 (29.7)	10.2 (30.3)
20–24	26.6 (44.2)	25.9 (43.8)
25–29	21.9 (41.4)	21.9 (41.4)
30–34	17.3 (37.8)	17.2 (37.8)
≥35	16.6 (37.2)	16.1 (36.7)
Birth order, % (SD)		
1st	33.5 (47.2)	34.1 (47.4)
2nd	20.4 (40.3)	20.7 (40.5)
3rd	14.6 (35.3)	14.3 (35.0)
4th	11.1 (31.5)	11.0 (31.3)
5th	8.2 (27.5)	7.8 (26.8)
6th	5.7 (23.3)	5.5 (22.9)
7th or later	6.4 (24.4)	6.6 (24.9)
DTP vaccination at 12 months of age, % (SD)		
0 DTP doses	12.4 (33.0)	10.1 (30.1)
1 DTP dose	3.3 (17.9)	2.8 (16.5)
2 DTP doses	5.3 (22.4)	4.6 (20.9)
3 DTP doses	79.0 (40.7)	82.6 (37.9)

Due to rounding, not all percentages add up to 100%. SD = standard deviation; DTP = diphtheria-tetanus-pertussis vaccine.

a Age at start of the school year in which the latest available household interview was conducted.

**Table 2:** Mother fixed-effects regressions of school grade attainment on measles vaccination status and control variables.

Variables	(1)	(2)	(3)	(4)
	Complete-case analysis without DTP3 covariate: beta-coefficient estimate (95% CI) *p*-value	Complete-case analysis with DTP3 covariate: beta-coefficient estimate (95% CI) *p*-value	Multiple imputation without DTP3 covariate: beta-coefficient estimate (95% CI) *p*-value	Multiple imputation with DTP3 covariate: beta-coefficient estimate (95% CI) *p*-value
(Reference category: no measles vaccination)				
Measles vaccination	0.188 (0.0424, 0.334) *p* = 0.011	0.188 (0.0197, 0.355) *p* = 0.029	0.149 (0.0419, 0.257) *p* = 0.006	0.143 (0.0108, 0.276) *p* = 0.034
Female	0.314 (0.194, 0.434) *p* < 0.001	0.315 (0.195, 0.436) *p* < 0.001	0.282 (0.192, 0.371) *p* < 0.001	0.282 (0.192, 0.371) *p* < 0.001
(Reference category: 8 years old^a^)				
6 years old	−0.893 (−1.396, −0.390) *p* = 0.001	−0.900 (−1.399, −0.400) *p* < 0.001	−1.217 (−1.595, −0.839) *p* < 0.001	−1.220 (−1.598, −0.841) *p* < 0.001
7 years old	−0.447 (−0.758, −0.136) *p* = 0.005	−0.442 (−0.755, −0.130) *p* = 0.006	−0.662 (−0.895, −0.428) *p* < 0.001	−0.662 (−0.896, −0.428) *p* < 0.001
9 years old	0.914 (0.605, 1.223) *p* < 0.001	0.900 (0.587, 1.213) *p* < 0.001	0.795 (0.550, 1.041) *p* < 0.001	0.792 (0.545, 1.038) *p* < 0.001
10 years old	1.624 (1.191, 2.057) *p* < 0.001	1.627 (1.194, 2.060) *p* < 0.001	1.727 (1.369, 2.084) *p* < 0.001	1.729 (1.370, 2.087) *p* < 0.001
11 years old	2.450 (1.863, 3.038) *p* < 0.001	2.435 (1.845, 3.025) *p* < 0.001	2.283 (1.764, 2.802) *p* < 0.001	2.283 (1.763, 2.803) *p* < 0.001
(Reference category: Born in 1997)				
Born in 1995	−0.227 (−0.710, 0.257) *p* = 0.358	−0.222 (−0.705, 0.262) *p* = 0.369	−0.0866 (−0.497, 0.323) *p* = 0.679	−0.0885 (−0.499, 0.322) *p* = 0.672
Born in 1996	−0.0718 (−0.428, 0.284) *p* = 0.693	−0.0803 (−0.436, 0.275) *p* = 0.658	−0.174 (−0.437, 0.0890) *p* = 0.194	−0.178 (−0.442, 0.0864) *p* = 0.187
Born in 1998	0.268 (−0.0426, 0.580) *p* = 0.091	0.265 (−0.0504, 0.580) *p* = 0.100	0.0822 (−0.161, 0.326) *p* = 0.508	0.0810 (−0.164, 0.326) *p* = 0.517
Born in 1999	0.132 (−0.322, 0.586) *p* = 0.568	0.116 (−0.346, 0.578) *p* = 0.622	0.104 (−0.243, 0.451) *p* = 0.557	0.0989 (−0.250, 0.448) *p* = 0.578
Born in 2000	−0.0387 (−0.715, 0.638) *p* = 0.911	−0.0397 (−0.717, 0.638) *p* = 0.909	0.0330 (−0.484, 0.550) *p* = 0.900	0.0330 (−0.484, 0.550) *p* = 0.901
(Reference category: Mother's age at birth: 20–24 years)				
<18 years	−0.134 (−0.639, 0.371) *p* = 0.603	−0.142 (−0.654, 0.370) *p* = 0.586	−0.187 (−0.565, 0.191) *p* = 0.331	−0.189 (−0.567, 0.190) *p* = 0.328
18–19 years	0.121 (−0.228, 0.469) *p* = 0.496	0.107 (−0.246, 0.459) *p* = 0.554	−0.0366 (−0.288, 0.215) *p* = 0.776	−0.0384 (−0.291, 0.214) *p* = 0.766
25–29 years	0.0281 (−0.231, 0.287) *p* = 0.831	0.0327 (−0.226, 0.292) *p* = 0.805	−0.0903 (−0.304, 0.124) *p* = 0.408	−0.0864 (−0.300, 0.127) *p* = 0.428
30–34 years	−0.116 (−0.528, 0.296) *p* = 0.582	−0.121 (−0.530, 0.288) *p* = 0.562	−0.237 (−0.556, 0.0821) *p* = 0.145	−0.235 (−0.554, 0.0840) *p* = 0.149
≥35 years	−0.407 (−1.036, 0.221) *p* = 0.204	−0.392 (−1.019, 0.235) *p* = 0.221	−0.503 (−0.977, −0.0282) *p* = 0.038	−0.496 (−0.970, −0.0213) *p* = 0.041
(Reference category: Birth order: 1st child)				
2nd child	−0.478 (−0.740, −0.216) *p* < 0.001	−0.479 (−0.741, −0.216) *p* < 0.001	−0.307 (−0.486, −0.128) *p* = 0.001	−0.307 (−0.486, −0.128) *p* = 0.001
3rd child	−0.766 (−1.232, −0.301) *p* = 0.001	−0.773 (−1.238, −0.307) *p* = 0.001	−0.445 (−0.740, −0.150) *p* = 0.003	−0.445 (−0.741, −0.149) *p* = 0.003
4th child	−1.302 (−1.931, −0.673) *p* < 0.001	−1.319 (−1.949, −0.688) *p* < 0.001	−0.636 (−1.032, −0.241) *p* = 0.002	−0.636 (−1.033, −0.240) *p* = 0.002
5th child	−1.701 (−2.488, −0.914) *p* < 0.001	−1.721 (−2.509, −0.933) *p* < 0.001	−0.821 (−1.317, −0.326) *p* = 0.001	−0.825 (−1.323, −0.326) *p* = 0.001
6th child	−2.071 (−3.022, −1.121) *p* < 0.001	−2.084 (−3.034, −1.134) *p* < 0.001	−0.871 (−1.462, −0.280) *p* = 0.004	−0.870 (−1.464, −0.277) *p* = 0.004
7th child or later	−2.495 (−3.645, −1.346) *p* < 0.001	−2.511 (−3.659, −1.363) *p* < 0.001	−0.994 (−1.695, −0.292) *p* = 0.006	−0.990 (−1.694, −0.286) *p* = 0.006
(Reference category: 3 DTP doses)				
0 DTP doses		−0.0219 (−0.259, 0.216) *p* = 0.857		−0.00176 (−0.211, 0.208) *p* = 0.987
1 DTP doses		0.282 (−0.0953, 0.659) *p* = 0.143		0.0676 (−0.268, 0.403) *p* = 0.693
2 DTP doses		−0.170 (−0.478, 0.137) *p* = 0.277		−0.107 (−0.369, 0.155) *p* = 0.422
Adjusted *R*^2^	0.743	0.743	0.618	0.618
Number of children	4783	4783	7509	7509
Number of mothers	4080	4080	6148	6148
Number of children with measles vaccination-discordant siblings	607	607	1031	1031

CI = confidence interval; DTP = diphtheria-tetanus-pertussis vaccine.

a Age at start of the school year in which the household interview was conducted.
